# 758. PICC Your Poison: Resident Beliefs and Attitudes Regarding Discharge Parenteral Antibiotics for Patients Who Inject Drugs

**DOI:** 10.1093/ofid/ofad500.819

**Published:** 2023-11-27

**Authors:** Scott Fabricant, Erika Abramson, Shashi Kapadia

**Affiliations:** New York-Presbyterian, New York, New York; Weill Cornell Medicine, New York, New York; Weill Cornell Medical Center, New York, New York

## Abstract

**Background:**

Antibiotic management of serious injection-related infections in patients who inject drugs (SIRI) can be challenging, partly due to provider concerns over negative outcomes related to peripherally inserted central catheter (PICC) use. This may lead to prolonged hospitalizations or premature discharges. Inpatient initiation of medication for opioid use disorder (MOUD) is becoming more common, helping mitigate negative outcomes. Because internal medicine (IM) residents are often the frontline providers in academic centers, it is important to understand their perspectives on SIRI care to improve outcomes.

**Methods:**

We surveyed IM residents in a large urban multi-center hospital system about SIRI care with a novel case-based survey that elicited management preferences, comfort, experience, and stigma. We gathered validity evidence for the instrument through use of existing published instruments, expert review, cognitive interviewing, and pilot testing. Results are reported using descriptive statistics and t-tests between groups.

**Results:**

Of 78 residents (response rate 39%), 66% were moderately or very uncomfortable discharging a patient with active substance use home with a PICC, while 12% were uncomfortable discharging with a PICC to a skilled nursing facility (SNF). About half believed institutional policies against PICC use in SIRI may act as barriers to discharge (Fig 1). Many residents were concerned about line misuse or infection, as well as home environment and follow-up (Fig 2). 79% felt initiation of MOUD while inpatient would increase their comfort with discharge with a PICC, with most willing to start patients on MOUD with expert assistance (Fig 3). Respondents named inexperience and difficulty connecting to outpatient providers, especially methadone clinics, as barriers to MOUD. Higher stigma was marginally associated with preference for oral antibiotics (p=0.07), but not with concerns over PICC-related outcomes or comfort discharging home with a PICC.

**Figure 1:**
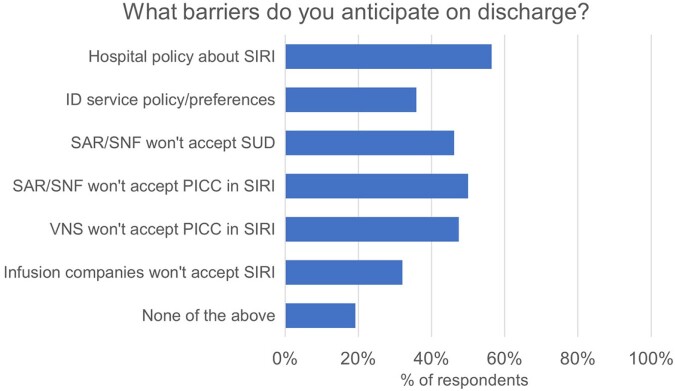
Perceived barriers to discharging patients with SIRI with a PICC. Abbreviations: SIRI: serious injection-related infections in people who inject drugs; PICC: peripherally inserted central catheter; SAR/SNF: subacute rehabilitation/skilled nursing facility; VNS: visiting nurse service

**Figure 2:**
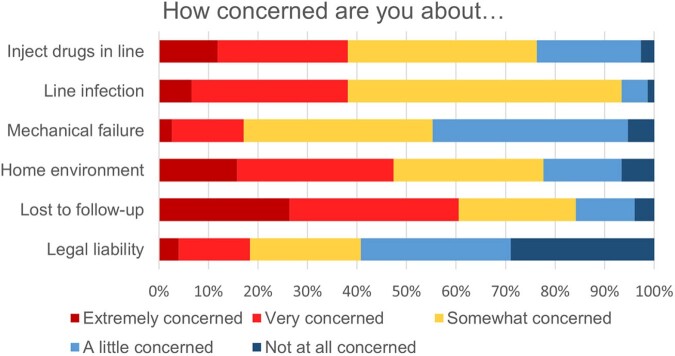
Cumulative percentage of respondents’ level of concern for various negative outcomes related to using peripherally inserted central catheters in patients who inject drugs.

**Figure 3:**
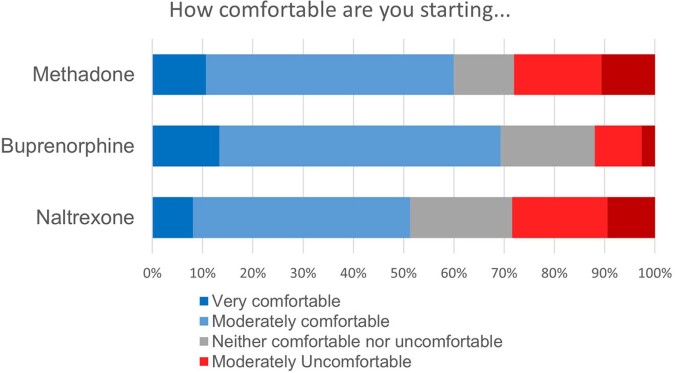
Cumulative percentage of respondents’ comfort level starting different types of medication for opioid use disorder (MOUD/MAT) with expert assistance.

**Conclusion:**

Most residents endorsed high levels of concern about PICC use for SIRI, related to patient outcomes and perceived institutional barriers, but identified MOUD as a mitigating factor. There is considerable room for educational and structural interventions to optimize care for patients with SIRI.

**Disclosures:**

**All Authors**: No reported disclosures

